# Optic Nerve Regeneration in Diabetic Retinopathy: Potentials and Challenges Ahead

**DOI:** 10.3390/ijms24021447

**Published:** 2023-01-11

**Authors:** Suqian Wu, Xiaofen Mo

**Affiliations:** Shanghai Key Laboratory of Visual Impairment and Restoration, Department of Ophthalmology and Vision Science, Eye & ENT Hospital, Fudan University, Shanghai 200031, China

**Keywords:** retinal ganglion cell, diabetic retinopathy, axon regeneration, optic nerve crush

## Abstract

Diabetic retinopathy (DR), the most common microvascular compilation of diabetes, is the leading cause of vision loss and blindness worldwide. Recent studies indicate that retinal neuron impairment occurs before any noticeable vascular changes in DR, and retinal ganglion cell (RGC) degeneration is one of the earliest signs. Axons of RGCs have little capacity to regenerate after injury, clinically leading the visual functional defects to become irreversible. In the past two decades, tremendous progress has been achieved to enable RGC axon regeneration in animal models of optic nerve injury, which holds promise for neural repair and visual restoration in DR. This review summarizes these advances and discusses the potential and challenges for developing optic nerve regeneration strategies treating DR.

## 1. Introduction

Diabetic retinopathy (DR) occurs in almost one-third of diabetes mellitus (DM) patients [1] and is the third most common complication and the most common microvascular complication of diabetes [2]. Although most of the time DR is clinically classified as a retinal vasculopathy, evidence of diabetic retinal neurodegeneration in patients, animal models and post-mortem human retinas has been continuously reported over the past 100 years [3,4,5,6]. Recent studies utilizing optic coherence tomography (OCT) [7,8,9] and electroretinogram (ERG) [10,11] indicate that diabetic neurodegeneration usually occurs before any visible retinal lesions. Thus, diabetic neurodegeneration is now considered a critical contributor to, instead of a major result of, diabetic retinovascular impairment [12,13].

Retinal ganglion cell (RGC) death is one of the earliest signs of diabetic retinal neuropathy, and diabetic optic nerve damage even precedes retinal abnormality [11,14]. The axons of RGCs course through the optic nerve and transmit visual information from the eye to the brain and are highly vulnerable to external insults, mostly because of their trajectory and space constraints. In streptozotocin (STZ)-induced diabetic rats, a reduced axon number and diameter in the optic nerve tract were both observed in the early stage of DM with morphologically unaffected RGCs and superior colliculus, suggesting optic nerve degeneration, the first structural impairment in the diabetic visual pathway [15]. As a part of the central nervous system (CNS), adult mammalian RGCs cannot proliferate or regrow, and their axons have little capacity to regenerate following damage, which in clinics leads patients to irreversible vision loss. In the past few decades, tremendous study progress has been made in elucidating mechanisms mediating RGC survival using animal models of DM [16,17,18,19] and in finding regulators of axon regeneration in models of optic nerve injury [20,21,22]. In this review, we discuss potential factors modulating optic nerve regeneration strategies in DR by drawing references from studies using optic nerve injury models.

## 2. RGC Degeneration in DR

**Figure 1:**
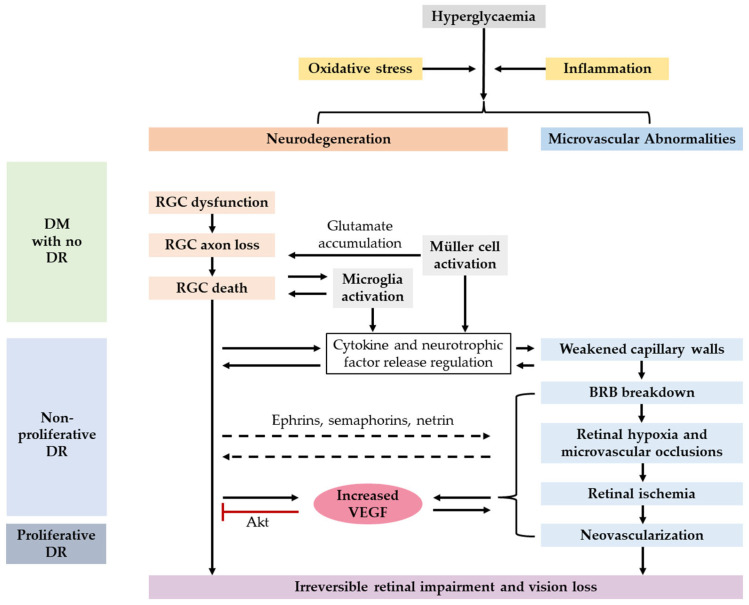
General phases of diabetic retinal neurodegeneration and microvascular abnormalities, and the controversial role of VEGF. DM, diabetes mellitus; DR, diabetic retinopathy; RGC, retinal ganglion cell; VEGF, vascular endothelial growth factor; BRB, blood-retinal barrier.

Although the exact pathogenesis of RGC loss in DR is still unclear, recent studies have already found a number of clues mediating this process, reviewed by Potilinski et al. and Soni et al. [16,17]. In brief, RGC apoptosis occurs in the early stage of DR, induced primarily by oxidative stress, extracellular glutamate accumulation and expression alterations of cytokines and neurotrophic factors (Figure 1) [19]. In addition, ATP-gated cation channels, such as the P2X7 receptor (P2X7R), may also be the targets for RGC death after early-phase injury [23]. In STZ-injected rats, RGC apoptosis, as well as a decreased mRNA level of manganese superoxidase dismutase (MnSOD), a critical antioxidant enzyme, was observed in the retina only four weeks after DM induction [24]. Another example of early neuronal injury is that receptor of advanced glycation end-products (RAGE), a neuronal injury-associated cytokine receptor, is upregulated in RGCs of high-fat-diet diabetic rats. In the later stages of DR, more RGCs are killed by the aforementioned factors.

The contributions of neurotrophic factors and vascular endothelial growth factor (VEGF) to diabetic RGC degeneration are somehow controversial [25]. For instance, brain-derived neurotrophic factor (BNDF) can upregulate insulin activity in diabetic animals [26] and is normally expressed in RGCs and other retinal cells. In diabetic patients, a decreased expression of BDNF in both serum and aqueous humor is found before any DR sign appears [27]. BDNF displays a neuroprotective effect in the early phase of DR but might trigger inflammation-related neural damage in later stages [28]. Likewise, VEGF is neuroprotective in the early DR phase, as inhibition of VEGF promotes RGC apoptosis in DR through enhancing Akt phosphorylation [29]. Nonetheless, VEGF is known to promote diabetic neovascularization, which may result in neurodegeneration, and therefore anti-VEGF administration is vital for treating proliferative DR. Thus, the impact of anti-VEGF drugs on RGC in DR requires further investigation.

## 3. Potential Optic Nerve Regeneration Approaches: Referenced from Optic Nerve Injury Models

Although many reports have found strategies to prevent RGC loss, few studies investigated any pro-regeneration approaches in DR. The main bottleneck lies in the current animal models, as most of the DR models fail to display an extensive axon loss in the optic nerve [30]. For example, 10-month STZ induction in adult mice causes just mild RGC density decrease and minimally reduced axons. On the other hand, optic nerve injury models can cause complete axon loss in the optic nerve as well as inflammatory responses in the retina and therefore are highly suitable for regeneration studies. Among all the optic nerve injury models, the most commonly used one is a crush injury, which destroys the axons in the optic nerve tract and leaves the dura and vessels intact. Using this model, tremendous progress has been accomplished in understanding the mechanisms regulating axon regeneration of degenerated RGCs, which provides a remarkable value for potential approaches to regenerating optic nerve in DR (Table 1).

### 3.1. Extrinsic Regulators of Optic Nerve Regeneration

Perhaps one of the evergreen questions in the field of neural regeneration research is why peripheral nerves can readily regenerate, whereas CNS nerves cannot regenerate after injury. A series of pioneer studies trying to answer this question found that RGCs could regenerate axons through a peripheral nerve environment [44,45,46], which led to a hypothesis that the CNS microenvironment is inhibitory toward axon regeneration.

One major suppressor of axon regeneration found in CNS is myelin [47]. Myelin is derived from oligodendrocytes and affects a series of inhibitory molecules, including myelin-associated glycoprotein, oligodendrocyte-myelin glycoprotein and Reticulon-4 (Nogo). All three molecules signal through the Nogo receptor (NgR) and paired immunoglobulin-like receptor B (PirB) [48]. In DR, Nogo-B is regarded as an angiogenesis enhancer [49], and NgR upregulation results in RGC apoptosis [50]. In contrast, Nogo-B knockdown partially decreases the permeability of retinal blood vessels under DR conditions. In the optic nerve crush model, intravitreal injection of a dominant-negative NgR [51,52], genetic deletion of NgR [53] and elimination of the myelin inhibitory signaling cascade via ROCK inhibitors [54] all receive just weak effects of axon regeneration. However, this effect could be enhanced if retinal inflammation induced by lens injury is present [52]. Therefore, retinal inflammation may be another critical factor regulating axon regeneration.

### 3.2. Inflammation-related Neurotrophic Factors

Berry and his colleagues [45] engrafted an autologous peripheral nerve fragment into the rat intravitreal space after optic nerve crush and found a great number of regenerating RGC axons. This effect was much reduced when grafts were freeze-and-thawed, suggesting the pro-regenerative contribution of living, inflammation-modulating Schwann cells in the peripheral nerve. Thus, manipulating proper inflammation-related factors may be a promising way for axon regeneration.

Neurotrophic factors, such as BDNF, pigmented epithelium-derived factor (PEDF), ciliary neurotrophic factor (CNTF) and insulin growth factor-1 (IGF-1), have been proposed to mediate effects of inflammation in the retina after axon impairment [25]. BDNF, produced by neurons, glial cells and pigmented epithelial cells in the retina [28,55,56], has been observed to have a reduced expression in DR [57]. Enhancing BDNF expression by either exogenous supply or gene overexpression has been demonstrated to promote RGC survival in STZ-induced diabetic mice [58,59]. However, based on the abundant study results from the optic nerve crush model, BDNF has been found to have minimal effect on RGC axon regeneration [60]. Likewise, significant RGC-protective but mild axon-regenerative abilities have also been observed in the case of CNTF [61], PEDF [62,63] and IGF-1 [64,65,66] administration. The underlying mechanism of the two distinct effects still remains unknown. One possible explanation is that neurotrophic factors trigger or/and are involved in intricate inflammatory responses regulating a large number of signaling pathways, which might counteract each other regarding axon regeneration. For example, although IGF-1 is an insulin analog and plays a neuroprotective role, it also upregulates VEGF in DR [67], enhancing the disease severity.

Taken together, these results suggest that, apart from the environmental inhibitory effect in CNS, matured RGC themselves also have little inherent ability to regrow their axons after injury. Thus, finding and then manipulating cell-intrinsic, regeneration-suppressing molecules among neurotrophic factor-associated signaling pathways appears to be a fruitful approach.

### 3.3. Intrinsic Repressors of Regeneration

IGF-1 downregulates the expression of phosphatase and tensin homolog deleted on Chromosome 10 (PTEN) [68], a tumor suppressor. Genetic knockout of PTEN induces robust axon regeneration of RGC in the optic nerve crush model [69,70]. This effect can be amplified along with overexpression of Sry-related high-mobility-box 11 (SOX11) [71], a key player controlling RGC development [72]. PTEN inhibition, as well as IGF-1 overexpression, activates PI3K/mTOR pathway, which may play a key role in promoting regeneration [70]. One supporting evidence is that treating with rapamycin, an mTOR inhibitor, negatively regulated IGF-1 expression [73] and also eliminated the axon regeneration led by *pten* deletion [69]. When combining *pten* deletion with cAMP and zymosan, which may upregulate CNTF [74], some of the axons are able to regenerate across the chiasm [75] and even reach central visual target areas [76].

Given its systematic impact on enhancing insulin activity, PTEN inhibition implies a hopeful therapeutic strategy for treating DM [77]. In the diabetic peripheral nerve, PTEN knockdown has already been shown to enhance myelinated axon regeneration after injury [78]. In a recent study using microRNA-26a-5p to protect RGC survival in the STZ-induced diabetic mouse model, a decreased PTEN expression was observed [31], suggesting that PTEN inhibition may be neuroprotective against DR.

CNTF upregulates cytokine signaling 3 (SOCS3) expression, inhibiting the JAK/STAT pathway [79]. Deletion of *socs3* enables RGCs to regenerate axons following optic nerve injury [80]. In addition, the combined deletion of *socs3* and *pten* promotes more sustained axon regeneration than each of those alone [81]. Moreover, adding this combination with CNTF and overexpression of *c-myc*, a proto-oncogene, led some of the axons to regenerate even across the optic chiasm and up to the ipsilateral and contralateral optic tracts [82].

Recent studies suggest that SOCS3 appears to protect against DR progression. SOCS3 acts as a repressor of matrix metalloproteinase 9 (MMP-9) in retinal microglia [32], and MMP-9 gene knockout prevented DR development in the STZ-induced mice [41]. On the other hand, genetic abrogation of SOCS3 in myeloid cells enhances the DR severity [33]. Besides, activation of the JAK/STAT signaling pathway is involved in DR pathogenesis [83,84], and suppressing this pathway inhibits retinal microvascular endothelial cell injury during DR progression [85]. Therefore, the impact of *socs3* knockout in RGCs under DR conditions remains unknown. Furthermore, whether there will be a synergistic or counteracting effect on RGC survival and axon regeneration in DR between *socs3* deletion and *pten* knockout deserves to be further investigated.

### 3.4. Intrinsic Regulators during RGC Development

Comparing gene expression between mature (postnatal) and developing (late embryonic) RGCs arises another way to identify regeneration-regulating components, given that the late embryonic RGCs still keep the axon-regenerative ability. In addition to SOX11 mentioned earlier, Krüppel-like factor (KLF) transcription factors act both positive and negative roles in RGC axon growth. In cell culture, KLF-6 and -7 promote, whereas KLF-4 and -9 repress axon growth [34]. Genetic deletion of KLF-4 leads to axon regeneration following optic nerve injury in vivo [34]. This effect can be enhanced in combination with CNTF administration and *socs3* knockout [35]. In addition, Galvao et al. identified the protein phosphatase dual specificity phosphatase 14 (DUSP14), a potential downstream target of KLF-4, as a regeneration repressor [86]. Interestingly, although shRNA-mediated *dusp14* knockdown induced regeneration after optic nerve crush, genetic knockout failed [86], suggesting a critical need to maintain baseline activities of some intrinsic developmental regulators even after RGCs mature.

In a recent study, forced overexpression of three Yamanaka stem cell factors, KLF-4, SOX2 and octamer-binding transcription factor 4 (OCT4), induced global DNA demethylation and successful axon regeneration, with some axons extending to the chiasm [87] after optic nerve crush. On the other hand, it is surprising to see consistent axon regenerative effects induced by opposing expression regulation of KLF-4, alone or with other transcription factors, in the same optic nerve injury model. Therefore, the underlying mechanism of these seemingly contradictory impacts needs further investigation.

KLF-4 is believed to participate in DM pathogenesis. It activates the JAK/STAT pathway [34,35] and VEGF signaling [36], therefore potentially enhancing the severity of the symptom. In addition, *dusp14* knockdown increases pancreatic beta-cell proliferation, which is likely to attenuate DM progression [37]. Hence, the effects of KLF-4 and DUSP14 inhibition on RGC survival and axon regeneration in DR models merit further studies.

Mitochondrial dynamics is also believed as an important contributor to neuron development and regenerative capacity. A recent study shows that intravitreal injection of M1, a molecule regulating axon mitochondrial dynamics, induced sustained axon regeneration in the mouse optic nerve crush model. Some regenerated axons extended through the optic chiasm and reached multiple subcortical areas. Moreover, the pupil light response was restored at six weeks post-crush [88]. This study suggests that, in addition to the aforementioned combinations of pro-regenerative treatments, long-distance axon regeneration can also be induced by a single molecule.

### 3.5. Ion Channel Regulators

The contribution of multiple ions to regeneration failure has gained attention in the past five years. For example, elevated level of zinc (Zn^2+^) in RGCs was associated with neuronal apoptosis after optic nerve crush [89]. In a study from the lab that identified M1, as we just mentioned, Au et al. characterized two Food and Drug Administration (FDA)-approved small-molecules, mexiletine and glycopyrrolate, that both displayed robust pro-regenerative capacity [90]. Mexiletine is a sodium channel blocker, and glycopyrrolate is a muscarinic acetylcholine receptor (mAChR) antagonist, which might subsequently inhibit Zn^2+^ uptake. Compared with mexiletine administration, weekly intravitreal injection of glycopyrrolate induced longer-distance RGC axon regeneration after optic nerve crush. Those regenerated axons extended across the chiasm and reached some visual targets, such as the olivary pretectal nucleus (OPN) in the midbrain, restoring pupil light response at six weeks post-crush.

Glycopyrrolate has been approved to treat peptic ulcers and is also reported to alleviate diabetic gustatory sweating [38]. In the case of modulating neural growth, however, more studies are needed.

### 3.6. Regeneration from Reprogrammed Müller Glia

There is a long-held saying in the study field of neural regeneration that is, “The colder the blood, the better the regeneration” [91]. In the context of RGC axon regeneration, it means that the rodent retina fails to self-heal, while some cold-blood species, such as zebrafish, keep a remarkable capacity to regenerate the retina and restore vision after injury. In the past two decades, a number of studies have indicated that Müller glial cells are the primary intrinsic source of regenerated neurons in fish [92,93] and therefore are regarded as the “retinal stem cells”. In zebrafish, a transition from quiescence to the reactive state in reprogrammed Müller glia was found, whereas, in mouse Müller glia, cell competence of proliferation and neurogenesis were repressed [94].

During the past 10 years, some in vivo studies have successfully transferred Müller glial cells into retinal neurons such as photoreceptors and bipolar cells [95,96,97]. A recent study using the optic nerve crush model shows that overexpression of two transcriptional factors, Brn3b and Math5, in Müller glial cells reprograms the cells into functional RGCs that regenerate axons even into certain brain targets and improve visual function [98]. Of note, these two molecules were not oncogenes, and no tumor suppressors were manually inhibited, which suggests the safety of its potential clinical application in the future.

A number of studies indicate Müller glia as a crucial modulator in DR pathogenesis. The neurotrophic factors and cytokines released by Müller glia contribute to detrimental effects on the microvasculature and simultaneously may have neuroprotective effects in DR [99]. Consequently, it is rather difficult to speculate whether reprogramming the Müller glia using the aforementioned method is therapeutic in DR without further validations under DR models.

### 3.7. RGC Subtype Specificity and Axon Regeneration

One consistent finding in those studies that successfully induced regeneration is that not all living RGCs displayed regenerative capacities upon stimulation. The reason for this regeneration preference may lie in the issue of RGC subtype specificity. Although RGCs share similar origins and morphology, they can be divided into various subtypes, e.g., at least 45 in mice [100,101], by different transcriptome expression spectrums and a wide range of light responses [102]. Potential preference for survival and axon regeneration of certain RGC subtypes has already been reported in human glaucoma patients [103] and mouse optic nerve crush models [104]. In the recent five years, the emergence of single-cell transcriptome sequencing led to a better understanding of RGC subtype specificity [100,105,106].

One RGC subtype that has been comprehensively studied is intrinsically photosensitive RGCs (ipRGCs), the only subtype that is able to directly respond to light through the presence of melanopsin. Recent data from single-cell sequencing indicate that ipRGC is one of the best surviving RGC subtypes following optic nerve crush [100]. Overexpression of melanopsin, the marker of ipRGCs, promotes RGC survival and axon regeneration by up-regulating mTOR complex 1 (mTORC1) [107]. Nevertheless, it has not been determined whether melanopsin overexpression enhanced the inherent re-growth capacity of existing ipRGCs or remodeled other RGC subtypes into ipRGC-like phenotypes. Through transcriptional screening of genes uniquely upregulated in ipRGCs, thrombospondin-1 (TSP-1), a downstream effector of CNTF, was found to boost axon regeneration when overexpressed in RGCs or Müller glia [108].

In STZ-induced diabetic mouse and rat models, preservation of ipRGCs was observed, suggesting the damage-resistant gift of this subtype in DR [109,110]. In human patients with non-proliferative diabetic retinopathy, the ipRGC function seems unaffected, determined by the melanopsin-mediated post-illumination pupillary light response (PIPR) tests [111]. However, in patients with advanced DR, cell loss and function impairment of ipRGCs were found [112,113]. TSP-1 is one of the earliest identified angiogenesis inhibitors and is downregulated in STZ-induced rats [114]. In addition, loss of TSP-1 exacerbates the DR pathogenesis, with increased acellular vessels [39]. Thus, it will be worthwhile to identify if overexpression of TSP-1 promotes RGC survival and preserves functioning axons in DR.

Another subtype that was determined injury-resilient is α-RGCs, making up the majority of PTEN-knockout RGCs that regenerate axons after damage [64,115]. Overexpression of osteopontin, one of the α-RGC markers, significantly boosted the axon re-growth among α-RGCs [64,105] after optic nerve crush. In DR, however, osteopontin is regarded as an angiogenesis-promoting factor, as it disrupts tight junctions among retinal vascular endothelial cells and increases vascular hyperpermeability [40].

### 3.8. Unveiling Regeneration Regulators from Multi-Omic Analyses

One major limitation of current strategies to identify regeneration modulators is the throughput of functional tests—usually one factor at a time. However, cutting-edge sequencing methods provide new chances for more comprehensive approaches to characterize gene expression atlas.

Single-cell RNA sequencing is a powerful tool for characterizing cell subtypes and can also be applied to identify survival- or even regeneration-regulating genes based on expression pattern changes of signature genes in disease models. Using this technique in the mouse optic nerve crush model, Tran and colleagues identified two new regeneration-promoting genes, urocortin (*Ucn*), tissue inhibitor of metalloproteinases 2 (*Timp2*), and two novel regeneration-suppressive genes, corticotropin-releasing hormone binding protein (*Crhbp*) and *Mmp9* [100]. Manipulating any of these four genes induced potent RGC axon regeneration. In DR pathogenesis, MMP-2 and -9 are both activated in the early stage and then destroy the mitochondria and augment retinal microvascular cell apoptosis, therefore promoting the breakdown of the blood–retinal barrier [116]. In the retina of the STZ-induced diabetic model, MMP-9 accumulates mostly in the nerve fiber layer and ganglion cell layer [117], and *Mmp9* knockout prevented DR development [41]. Thus, MMP-9 inhibition holds promise for neuroprotection and neuro-regeneration in DR and deserves further studies.

In two recent studies leveraging single-cell sequencing, transcriptome expression patterns were compared between that of regenerating RGCs and surviving-but-not-regenerative RGCs after optic nerve crush in a mouse model with PTEN-null RGCs [105,115]. Results from the two studies revealed a series of pro-regenerative genes, including seven newly-identified members: galanin (*Gal*), corticotropin-releasing-hormone (*Crh*), Wilms’ tumor 1 (*Wt1*), Annexin A2 (*Anxa2*), membrane palmitoylated protein 1 (*Mpp1*), Perilipin 2 (*Plin2*), and acetyl-coenzyme A acyltransferase 2 (*Acaa2*) [105]. Of note, three of the seven pro-regeneration genes are not tumorigenesis-related (*Crh*, *Mpp1* and *Plin2*), suggesting that tumor suppressor deletion-mediated regeneration, such as PTEN or/and SOCS3 knockout, might be substituted with safer downstream factors. In the STZ-induced model, CRH expression is upregulated in the retina, and knockdown of *Crh* enhances retinal inflammatory response and visual impairment, suggesting CRH has a protective role against DR progress [42]. Nevertheless, the exact contribution of CRH, as well as the other six factors in DR pathogenesis, requires further investigation.

Single-cell sequencing has also been applied to identify gene expression spectrum in the diabetic retina [118,119,120,121,122], and some reports showed transcriptional differences between normal and diabetic RGCs [120,122]. Further studies may be required to compare different gene expression patterns between injury-resilient and -susceptible RGCs after DR induction.

Recently, the application of in vivo CRISPR screening on mouse optic nerve crush models enables researchers to identify a great number of negative regulators of RGC axon regeneration. Tian et al. reported 13 transcription factors as regeneration suppressors after crush, confirmed by the results that knocking out any of those 13 genes in RGCs led to significant axon regeneration [123]. They are transcription factor 3 (TCF3), PRKC apoptosis WT1 regulator (PAWR), tumor growth factor-beta induced factor homeobox 1 (TGIF1), LIM Homeobox 6 (LHX6), retinoblastoma binding protein 7 (RBBP7), SIN3 transcription regulator family member A (SIN3A), E1A Binding Protein P300 (EP300), early B-cell factor 3 (EBF3), CCCTC-binding factor (CTCF), LIM homeobox gene 2 (LHX2), transcription factor 24 (TCF24), collaborator of the alternate reading frame (CARF) and stromal antigen 1 (STAG1). Seven of them are considered to be tumor suppressors, and four are oncogenes. Regarding DR pathogenesis, the contributions of these transcription factors need further investigation.

### 3.9. Promoting Regeneration by Modulating Intercellular Activities

In addition to direct manipulation of RGCs, regulating interactions between RGC and other retinal cells provides another path to axon regeneration. An excellent example is the regulation of *Lin28*, an oncogene. A previous study showed that Lin28 overexpression in RGCs promoted mild axon regeneration after optic nerve crush [124]. Later then, however, Zhang et al. reported that overexpression of Lin28 in amacrine cells, instead of RGCs, greatly amplified IGF-1-mediated RGC axon regeneration which used to be minimal [125]. Next, they found that optic nerve injury hyperactivated the amacrine cells and inhibited their synaptic activities. In addition, a large number of IGF-1 receptors (IGF-1Rs) enriched in the cilia of RGCs were lost after injury. Lin28 removed the amacrine cell synaptic inhibition and also prevented IGF-1R loss, which potentially potentiated the IGF-1-induced axon regeneration.

Lin28a/b overexpression in mouse myoblasts promotes an insulin-sensitized state that resists high-fat-diet induced DM, whereas mTOR inhibition abrogates Lin28a-dependent insulin sensitivity [43], suggesting that Lin28a functions, in part, through similar signaling pathways as PTEN knockdown does. Thus, we speculate that Lin28 may also be neuroprotective against DR.

## 4. Relationship between Survival and Regeneration

In the past two decades, a lot more studies that have shown successful protection of RGC survival through a variety of strategies, unfortunately, failed to induce robust axon regeneration after optic nerve crush, demonstrating that simply maintaining RGC viability is somehow insufficient for optic nerve regeneration [22,126]. Moreover, as mentioned earlier, even under some conditions that ensure RGC survival and long-distance axon regeneration, most surviving RGCs still do not regrow axons. For example, co-deletion of PTEN and SOCS3, accompanied by CNTF administration, led to axon regeneration from less than 10% of all living RGCs following optic nerve crush [115]. Gene ontology (GO) analysis showed that the axon-regenerating RGCs selectively expressed genes mostly related to manipulating cell migration/motility and cell adhesion, and, in contrast, those surviving-but-not-regenerating RGCs were enriched by genes related to synapse organization and neuronal differentiation [115]. However, the exact mechanism regulating regeneration in surviving RGCs remains to be uncovered.

Empirically, all successful pro-regenerative treatments also preserve RGCs after injury. However, this may not always be the case, according to some recent reports. In the aforementioned study of overexpressing three Yamanaka stem cell factors, no significantly larger number of surviving RGCs was observed in a microbead-induced mouse glaucoma model, although a reduced axon loss was suggested [87]. Another example is the case of SOX11 overexpression, where even fewer RGCs survived the optic nerve crush compared to untreated controls [71], although significant axon regeneration was induced. Conversely, the downregulation of SOX11 led to more living RGCs in the same animal model [127], whereas no axon regeneration was observed. Further investigation demonstrated that SOX11 overexpression kills α-RGCs, and protects the other subsets [71]. Moreover, when SOX11 was manipulated by mutating a single residue at lysine 91 (K91), its overexpression promoted a greater regeneration but further exacerbated the death of α-RGCs as well ipRGCs [128]. In the aforementioned study using in vivo CRISPR screen, only one of the 13 identified anti-regenerative factors, EBF3, is RGC-protective upon knockout [123]. Taken together, much more efforts still need to be made to understand more about the molecular mechanisms modulating RGC survival and axon regeneration.

## 5. Relationship between Axon Guidance and Angiogenesis

In addition to the distance of axon regeneration, another critical contributor to functional recovery after optic nerve injury is axon pathfinding: Do RGCs re-extend their axons back to the original terminals and form connections with appropriate targets? In fact, many papers that reported long-distance RGC axon regeneration also described axon misguidance errors, mostly at the optic chiasm. Some regenerated axons were observed coursing into the contralateral optic nerve [75,76,81,129,130], and some others even U-turned back towards the lesion site [129,130]. Therefore, studies of guidance cues during regeneration are of vital importance. Interestingly, similar pathfinding errors have also been reported in RGC axon development lacking certain guidance cues [131,132], which gave us a crucial reference.

Semaphorins, ephrin/Eph families and netrins have been regarded as major axon guidance molecules during axon development [131,133]. Of note, these cues also regulate vascular outgrowth and death, which play a key role in DR pathogenesis. RGC-derived Sema3A is upregulated in early DR and then induces vascular hyperpermeability through its receptor Neuropilin-1 (Nrp1), subsequently participating in blood-retinal barrier (BRB) breakdown [134]. Ojima and colleagues intravitreally injected ephrinA1 in a VEGF-induced rat neovascular retinopathy model resembling manifestations of proliferative DR and found that ephrinA1 inhibited retinal neovascularization and BRB breakdown [135]. The impact of netrins on angiogenesis remains seemingly contradictory, as netrin-1 and -4 have been reported as either pro- or anti-angiogenic, presumably depending on the receptors they bind [136]. In the STZ-induced rat DR model, Yu et al. intravitreally injected 0.1 μg/mL or 5 μg/mL netrin-1 and found the neovascularization effects were opposing: 0.1 μg/mL of netrin-1 increased the numbers of new retinal vessels, while 5 μg/mL of netrin-1 repressed retinal neovascularization [137]. In another study using the same model, Cao and colleagues intravitreally injected 2 μL of 100 μg/mL netrin-1 or -4 and showed that either of the treatments inhibited VEGF expression and suppressed retinal vascular leakage, suggesting the therapeutic potential of netrin-1 and netrin-4 [138]. Taken together, further studies are required for the proper regulation of the guidance cues that avoid misguiding regenerated axons and also suppress vasculopathy in DR.

## 6. Summary

In the past two decades, optic nerve regeneration, as a premier model of regenerative treatment in the CNS, has been successfully induced in numerous studies under the optic nerve injury models. Nonetheless, it should be noted that promoting RGC axon regeneration is a much more complex issue in the context of DR, and more questions remain to be answered. First, the exact mechanism of RGC degeneration in DR is not clear yet, and the association between RGC death and retinal microvascular injury needs further study. Second, the proper therapeutic window for regeneration treatment is still elusive. Considering the early onset of RGC malfunction in DR, performing function tests such as ERG at an early time point should be preferred, and immediate neuroprotective medical intervention may fight against diabetic RGC death [11]. Moreover, perhaps the most challenging task is the combination of regeneration induction and angiogenesis blockade, as some of the identified pro-regenerative factors, such as osteopontin, may have a counterforce in the form of DR progression, e.g., promoting vessel growth. Furthermore, considering the counteracting role of VEGF in DR progression, it would be interesting to study if those pro-regenerative strategies will enhance retinal vascular repairment, and a combination of neuroprotection and anti-neovascularization approach may be suggested. In basic research settings, the Cre-LoxP system might help with knocking out a targeted gene in selected cell type(s), and viral transfections with specific vector(s) and promoter(s) may enable targeting treatments. However, the safety and efficacy of these techniques require further validation in translational research settings.

The most crucial issue that limits the axon regenerative study in DR might be the lack of a suitable animal model. To deal with this short board, 1) it becomes increasingly important to monitor the functional improvement of retinal neurons via in vivo tests; for example, pattern ERG could be a useful tool to assess new drugs on RGC function [139]; and 2) combining classical DR induction with optic nerve injury may be an alternative approach, as similar combinations have already been used for years in studying peripheral nerve regeneration in diabetic neuropathy [140].

In addition to promoting endogenous RGC survival and axon regeneration, the area of RGC replacement, such as axon regeneration, has also advanced in recent years. Exogeneous primary RGCs or stem cell-derived RGC-like cells have been reported to survive, integrate into the host retinal circuity and present a certain extent of electrophysiological function after transplant [141,142,143,144]. However, it may be much more challenging to achieve a more advanced state of vision recovery, as visual function is usually shaped during early childhood. One major barrier is how to provide a better milieu that is suitable for the survival and integration of transplanted cells [145], and it will become even more complex when it comes to DR, in which neural and vascular systems are both damaged.

In short, optic nerve regeneration in DR, facing its potential and challenges ahead, is likely to keep fruitful for a long time as the progress is just started compared to regeneration studies in other diseases such as glaucoma and optic nerve trauma.

## Figures and Tables

**Table 1:** Reported DR-related findings of pro-regenerative targets under optic nerve injury models.

Target	Pro-Regenerative Application in Optic Nerve Injury Model	Reported Findings Associated with DR or DM Pathogenesis	References
PTEN	Genetic downregulation in RGCs	Decreased under microRNA-26a-5p-induced neuroprotection in STZ-induced mouse model	[31]
SOCS3	Genetic downregulation in RGCs	Deletion might enhance DR severity via MMP-9	[32,33]
KLF-4	Genetic downregulation in RGCs	Might participate in DR progression by activating JAK/STAT pathway and VEGF signaling	[34,35,36]
DUSP14	Genetic downregulation in RGCs	Knockdown increases pancreatic β-cell proliferation	[37]
Glycopyrrolate	Intravitreal injection	Reported to alleviate diabetic gustatory sweating	[38]
TSP-1	Genetic overexpression in RGCs	TSP-1 loss exacerbates the DR pathogenesis	[39]
Osteopontin	Genetic overexpression in RGCs	Angiogenesis-promoting factor in DR	[40]
MMP-9	Genetic downregulation in RGCs	Knockout prevented DR development in the STZ-induced mice	[41]
CRH	Genetic overexpression in RGCs	knockdown enhances retinal inflammatory response and visual impairment in STZ-induced model	[42]
Lin28	Genetic overexpression in amacrine cells (+ intravitreal injection of IGF-1)	overexpression in mouse myoblasts promotes an insulin-sensitized state that resists high-fat-diet induced DM	[43]

DR, Diabetic retinopathy; DM, diabetes mellitus; RGC, retinal ganglion cell; STZ, streptozotocin; MMP-9, metalloproteinase 9; VEGF, vascular endothelial growth factor; TSP-1, thrombospondin-1; CRH, corticotropin-releasing-hormone; IGF-1, insulin growth factor 1.

## References

[1] Lee R., Wong T.Y., Sabanayagam C. (2015). Epidemiology of diabetic retinopathy, diabetic macular edema and related vision loss. Eye Vis..

[2] UK Prospective Diabetes Study Group (1998). Tight blood pressure control and risk of macrovascular and microvascular complications in type 2 diabetes: Ukpds 38. BMJ.

[3] Wolter J.R. (1961). Diabetic retinopathy. Am. J. Ophthalmol..

[4] Simo R., Hernandez C., European Consortium for the Early Treatment of Diabetic Retinopathy (2014). Neurodegeneration in the diabetic eye: New insights and therapeutic perspectives. Trends Endocrinol. Metab..

[5] Antonetti D.A., Klein R., Gardner T.W. (2012). Diabetic retinopathy. N. Engl. J. Med..

[6] Mendonca H.R., Carpi-Santos R., da Costa Calaza K., Blanco Martinez A.M. (2020). Neuroinflammation and oxidative stress act in concert to promote neurodegeneration in the diabetic retina and optic nerve: Galectin-3 participation. Neural Regen. Res..

[7] Ng D.S., Chiang P.P., Tan G., Cheung C.G., Cheng C.Y., Cheung C.Y., Wong T.Y., Lamoureux E.L., Ikram M.K. (2016). Retinal ganglion cell neuronal damage in diabetes and diabetic retinopathy. Clin. Exp. Ophthalmol..

[8] Carpineto P., Toto L., Aloia R., Ciciarelli V., Borrelli E., Vitacolonna E., Di Nicola M., Di Antonio L., Mastropasqua R. (2016). Neuroretinal alterations in the early stages of diabetic retinopathy in patients with type 2 diabetes mellitus. Eye.

[9] Chhablani J., Sharma A., Goud A., Peguda H.K., Rao H.L., Begum V.U., Barteselli G. (2015). Neurodegeneration in type 2 diabetes: Evidence from spectral-domain optical coherence tomography. Investig. Ophthalmol. Vis. Sci..

[10] Falsini B., Porciatti V., Scalia G., Caputo S., Minnella A., Di Leo M.A., Ghirlanda G. (1989). Steady-state pattern electroretinogram in insulin-dependent diabetics with no or minimal retinopathy. Doc. Ophthalmol..

[11] Amato R., Catalani E., Dal Monte M., Cammalleri M., Cervia D., Casini G. (2022). Morpho-functional analysis of the early changes induced in retinal ganglion cells by the onset of diabetic retinopathy: The effects of a neuroprotective strategy. Pharmacol. Res..

[12] Sohn E.H., van Dijk H.W., Jiao C., Kok P.H., Jeong W., Demirkaya N., Garmager A., Wit F., Kucukevcilioglu M., van Velthoven M.E. (2016). Retinal neurodegeneration may precede microvascular changes characteristic of diabetic retinopathy in diabetes mellitus. Proc. Natl. Acad. Sci. USA.

[13] Jonsson K.B., Frydkjaer-Olsen U., Grauslund J. (2016). Vascular changes and neurodegeneration in the early stages of diabetic retinopathy: Which comes first?. Ophthalmic Res..

[14] Parisi V., Uccioli L., Monticone G., Parisi L., Manni G., Ippoliti D., Menzinger G., Bucci M.G. (1997). Electrophysiological assessment of visual function in iddm patients. Electroencephalogr. Clin. Neurophysiol..

[15] Fernandez D.C., Pasquini L.A., Dorfman D., Aldana Marcos H.J., Rosenstein R.E. (2012). Early distal axonopathy of the visual pathway in experimental diabetes. Am. J. Pathol..

[16] Soni D., Sagar P., Takkar B. (2021). Diabetic retinal neurodegeneration as a form of diabetic retinopathy. Int. Ophthalmol..

[17] Potilinski M.C., Lorenc V., Perisset S., Gallo J.E. (2020). Mechanisms behind retinal ganglion cell loss in diabetes and therapeutic approach. Int. J. Mol. Sci..

[18] Chakravarthy H., Devanathan V. (2018). Molecular mechanisms mediating diabetic retinal neurodegeneration: Potential research avenues and therapeutic targets. J. Mol. Neurosci..

[19] Oshitari T. (2021). The pathogenesis and therapeutic approaches of diabetic neuropathy in the retina. Int. J. Mol. Sci..

[20] Fague L., Liu Y.A., Marsh-Armstrong N. (2021). The basic science of optic nerve regeneration. Ann. Transl. Med..

[21] Laha B., Stafford B.K., Huberman A.D. (2017). Regenerating optic pathways from the eye to the brain. Science.

[22] Benowitz L.I., He Z., Goldberg J.L. (2017). Reaching the brain: Advances in optic nerve regeneration. Exp. Neurol..

[23] Platania C.B.M., Drago F., Bucolo C. (2022). The p2x7 receptor as a new pharmacological target for retinal diseases. Biochem. Pharmacol..

[24] Li X., Zhang M., Zhou H. (2014). The morphological features and mitochondrial oxidative stress mechanism of the retinal neurons apoptosis in early diabetic rats. J. Diabetes Res..

[25] Bikbova G., Oshitari T., Baba T., Yamamoto S. (2014). Neurotrophic factors for retinal ganglion cell neuropathy—With a special reference to diabetic neuropathy in the retina. Curr. Diabetes Rev..

[26] Hanyu O., Yamatani K., Ikarashi T., Soda S., Maruyama S., Kamimura T., Kaneko S., Hirayama S., Suzuki K., Nakagawa O. (2003). Brain-derived neurotrophic factor modulates glucagon secretion from pancreatic alpha cells: Its contribution to glucose metabolism. Diabetes Obes. Metab..

[27] Uzel A.G.T., UGurlu N., Toklu Y., ÇIçek M., Boral B., Sener B., ÇaGil N. (2020). Relationship between stages of diabetic retinopathy and levels of brain-derived neurotrophic factor in aqueous humor and serum. Retina.

[28] Afarid M., Namvar E., Sanie-Jahromi F. (2020). Diabetic retinopathy and bdnf: A review on its molecular basis and clinical applications. J. Ophthalmol..

[29] Park H.Y., Kim J.H., Park C.K. (2014). Neuronal cell death in the inner retina and the influence of vascular endothelial growth factor inhibition in a diabetic rat model. Am. J. Pathol..

[30] Howell S.J., Mekhail M.N., Azem R., Ward N.L., Kern T.S. (2013). Degeneration of retinal ganglion cells in diabetic dogs and mice: Relationship to glycemic control and retinal capillary degeneration. Mol. Vis..

[31] Shi R., Liu D.D., Cao Y., Xue Y.S. (2022). Microrna-26a-5p prevents retinal neuronal cell death in diabetic mice by targeting pten. Curr. Eye Res..

[32] Zhu S.H., Liu B.Q., Hao M.J., Fan Y.X., Qian C., Teng P., Zhou X.W., Hu L., Liu W.T., Yuan Z.L. (2017). Paeoniflorin suppressed high glucose-induced retinal microglia mmp-9 expression and inflammatory response via inhibition of tlr4/nf-kappab pathway through upregulation of socs3 in diabetic retinopathy. Inflammation.

[33] Chen M., Obasanmi G., Armstrong D., Lavery N.J., Kissenpfennig A., Lois N., Xu H. (2019). Stat3 activation in circulating myeloid-derived cells contributes to retinal microvascular dysfunction in diabetes. J. Neuroinflam..

[34] Moore D.L., Blackmore M.G., Hu Y., Kaestner K.H., Bixby J.L., Lemmon V.P., Goldberg J.L. (2009). Klf family members regulate intrinsic axon regeneration ability. Science.

[35] Qin S., Zou Y., Zhang C.L. (2013). Cross-talk between klf4 and stat3 regulates axon regeneration. Nat. Commun..

[36] Wang Y., Yang C., Gu Q., Sims M., Gu W., Pfeffer L.M., Yue J. (2015). Klf4 promotes angiogenesis by activating vegf signaling in human retinal microvascular endothelial cells. PLoS ONE.

[37] Klinger S., Poussin C., Debril M.B., Dolci W., Halban P.A., Thorens B. (2008). Increasing glp-1-induced beta-cell proliferation by silencing the negative regulators of signaling camp response element modulator-alpha and dusp14. Diabetes.

[38] Shaw J.E., Abbott C.A., Tindle K., Hollis S., Boulton A.J. (1997). A randomised controlled trial of topical glycopyrrolate, the first specific treatment for diabetic gustatory sweating. Diabetologia.

[39] Sorenson C.M., Wang S., Gendron R., Paradis H., Sheibani N. (2013). Thrombospondin-1 deficiency exacerbates the pathogenesis of diabetic retinopathy. J. Diabetes Metab..

[40] Someya H., Ito M., Nishio Y., Sato T., Harimoto K., Takeuchi M. (2022). Osteopontin-induced vascular hyperpermeability through tight junction disruption in diabetic retina. Exp. Eye Res..

[41] Kowluru R.A., Mohammad G., dos Santos J.M., Zhong Q. (2011). Abrogation of mmp-9 gene protects against the development of retinopathy in diabetic mice by preventing mitochondrial damage. Diabetes.

[42] Huang C., Zhu H.J., Li H., Li Q.X., Li F.M., Cheng L., Liu Y.G. (2018). P38-mapk pathway is activated in retinopathy of microvascular disease of stz-induced diabetic rat model. Eur. Rev. Med. Pharmacol. Sci..

[43] Zhu H., Shyh-Chang N., Segre A.V., Shinoda G., Shah S.P., Einhorn W.S., Takeuchi A., Engreitz J.M., Hagan J.P., Kharas M.G. (2011). The lin28/let-7 axis regulates glucose metabolism. Cell.

[44] Whiteley S.J., Sauve Y., Aviles-Trigueros M., Vidal-Sanz M., Lund R.D. (1998). Extent and duration of recovered pupillary light reflex following retinal ganglion cell axon regeneration through peripheral nerve grafts directed to the pretectum in adult rats. Exp. Neurol..

[45] Berry M., Carlile J., Hunter A. (1996). Peripheral nerve explants grafted into the vitreous body of the eye promote the regeneration of retinal ganglion cell axons severed in the optic nerve. J. Neurocytol..

[46] Caroni P., Savio T., Schwab M.E. (1988). Central nervous system regeneration: Oligodendrocytes and myelin as non-permissive substrates for neurite growth. Prog. Brain Res..

[47] Geoffroy C.G., Zheng B. (2014). Myelin-associated inhibitors in axonal growth after cns injury. Curr. Opin. Neurobiol..

[48] Yiu G., He Z. (2006). Glial inhibition of cns axon regeneration. Nat. Rev. Neurosci..

[49] Zhang Y., Wang L., Zhang Y., Wang M., Sun Q., Xia F., Wang R., Liu L. (2017). Nogo-b promotes angiogenesis in proliferative diabetic retinopathy via vegf/pi3k/akt pathway in an autocrine manner. Cell. Physiol. Biochem..

[50] Liu X., Zuo Z., Liu W., Wang Z., Hou Y., Fu Y., Han Y. (2014). Upregulation of nogo receptor expression induces apoptosis of retinal ganglion cells in diabetic rats. Neural Regen. Res..

[51] Wang X., Lin J., Arzeno A., Choi J.Y., Boccio J., Frieden E., Bhargava A., Maynard G., Tsai J.C., Strittmatter S.M. (2015). Intravitreal delivery of human ngr-fc decoy protein regenerates axons after optic nerve crush and protects ganglion cells in glaucoma models. Investig. Ophthalmol. Vis. Sci..

[52] Fischer D., He Z., Benowitz L.I. (2004). Counteracting the nogo receptor enhances optic nerve regeneration if retinal ganglion cells are in an active growth state. J. Neurosci..

[53] Dickendesher T.L., Baldwin K.T., Mironova Y.A., Koriyama Y., Raiker S.J., Askew K.L., Wood A., Geoffroy C.G., Zheng B., Liepmann C.D. (2012). Ngr1 and ngr3 are receptors for chondroitin sulfate proteoglycans. Nat. Neurosci..

[54] Lingor P., Tonges L., Pieper N., Bermel C., Barski E., Planchamp V., Bahr M. (2008). Rock inhibition and cntf interact on intrinsic signalling pathways and differentially regulate survival and regeneration in retinal ganglion cells. Brain.

[55] Ola M.S., Nawaz M.I., Khan H.A., Alhomida A.S. (2013). Neurodegeneration and neuroprotection in diabetic retinopathy. Int. J. Mol. Sci..

[56] Ming M., Li X., Fan X., Yang D., Li L., Chen S., Gu Q., Le W. (2009). Retinal pigment epithelial cells secrete neurotrophic factors and synthesize dopamine: Possible contribution to therapeutic effects of rpe cell transplantation in Parkinson’s disease. J. Transl. Med..

[57] Ola M.S., Nawaz M.I., El-Asrar A.A., Abouammoh M., Alhomida A.S. (2013). Reduced levels of brain derived neurotrophic factor (bdnf) in the serum of diabetic retinopathy patients and in the retina of diabetic rats. Cell. Mol. Neurobiol..

[58] Rong L., Gu X., Xie J., Zeng Y., Li Q., Chen S., Zou T., Xue L., Xu H., Yin Z.Q. (2018). Bone marrow cd133(+) stem cells ameliorate visual dysfunction in streptozotocin-induced diabetic mice with early diabetic retinopathy. Cell Transplant..

[59] Gong Y., Chang Z.P., Ren R.T., Wei S.H., Zhou H.F., Chen X.F., Hou B.K., Jin X., Zhang M.N. (2012). Protective effects of adeno-associated virus mediated brain-derived neurotrophic factor expression on retinal ganglion cells in diabetic rats. Cell. Mol. Neurobiol..

[60] Leaver S.G., Cui Q., Plant G.W., Arulpragasam A., Hisheh S., Verhaagen J., Harvey A.R. (2006). Aav-mediated expression of cntf promotes long-term survival and regeneration of adult rat retinal ganglion cells. Gene Ther..

[61] Weise J., Isenmann S., Klocker N., Kugler S., Hirsch S., Gravel C., Bahr M. (2000). Adenovirus-mediated expression of ciliary neurotrophic factor (cntf) rescues axotomized rat retinal ganglion cells but does not support axonal regeneration in vivo. Neurobiol. Dis..

[62] Vigneswara V., Berry M., Logan A., Ahmed Z. (2013). Pigment epithelium-derived factor is retinal ganglion cell neuroprotective and axogenic after optic nerve crush injury. Investig. Ophthalmol. Vis. Sci..

[63] Barnstable C.J., Tombran-Tink J. (2004). Neuroprotective and antiangiogenic actions of pedf in the eye: Molecular targets and therapeutic potential. Prog. Retin. Eye Res..

[64] Duan X., Qiao M., Bei F., Kim I.J., He Z., Sanes J.R. (2015). Subtype-specific regeneration of retinal ganglion cells following axotomy: Effects of osteopontin and mtor signaling. Neuron.

[65] Seigel G.M., Lupien S.B., Campbell L.M., Ishii D.N. (2006). Systemic igf-i treatment inhibits cell death in diabetic rat retina. J. Diabetes Complicat..

[66] Kermer P., Klocker N., Labes M., Bahr M. (2000). Insulin-like growth factor-i protects axotomized rat retinal ganglion cells from secondary death via pi3-k-dependent akt phosphorylation and inhibition of caspase-3 in vivo. J. Neurosci..

[67] Chantelau E., Kimmerle R., Meyer-Schwickerath R. (2008). Insulin, insulin analogues and diabetic retinopathy. Arch. Physiol. Biochem..

[68] Ma J., Sawai H., Matsuo Y., Ochi N., Yasuda A., Takahashi H., Wakasugi T., Funahashi H., Sato M., Takeyama H. (2010). Igf-1 mediates pten suppression and enhances cell invasion and proliferation via activation of the igf-1/pi3k/akt signaling pathway in pancreatic cancer cells. J. Surg. Res..

[69] Park K.K., Liu K., Hu Y., Smith P.D., Wang C., Cai B., Xu B., Connolly L., Kramvis I., Sahin M. (2008). Promoting axon regeneration in the adult cns by modulation of the pten/mtor pathway. Science.

[70] Zhang J., Yang D., Huang H., Sun Y., Hu Y. (2018). Coordination of necessary and permissive signals by pten inhibition for cns axon regeneration. Front. Neurosci..

[71] Norsworthy M.W., Bei F., Kawaguchi R., Wang Q., Tran N.M., Li Y., Brommer B., Zhang Y., Wang C., Sanes J.R. (2017). Sox11 expression promotes regeneration of some retinal ganglion cell types but kills others. Neuron.

[72] Jiang Y., Ding Q., Xie X., Libby R.T., Lefebvre V., Gan L. (2013). Transcription factors sox4 and sox11 function redundantly to regulate the development of mouse retinal ganglion cells. J. Biol. Chem..

[73] Bibollet-Bahena O., Almazan G. (2009). Igf-1-stimulated protein synthesis in oligodendrocyte progenitors requires pi3k/mtor/akt and mek/erk pathways. J. Neurochem..

[74] Muller A., Hauk T.G., Fischer D. (2007). Astrocyte-derived cntf switches mature rgcs to a regenerative state following inflammatory stimulation. Brain.

[75] Kurimoto T., Yin Y., Omura K., Gilbert H.Y., Kim D., Cen L.P., Moko L., Kugler S., Benowitz L.I. (2010). Long-distance axon regeneration in the mature optic nerve: Contributions of oncomodulin, camp, and pten gene deletion. J. Neurosci..

[76] De Lima S., Koriyama Y., Kurimoto T., Oliveira J.T., Yin Y., Li Y., Gilbert H.Y., Fagiolini M., Martinez A.M., Benowitz L. (2012). Full-length axon regeneration in the adult mouse optic nerve and partial recovery of simple visual behaviors. Proc. Natl. Acad. Sci. USA.

[77] Li Y.Z., Di Cristofano A., Woo M. (2020). Metabolic role of pten in insulin signaling and resistance. Cold Spring Harb. Perspect. Med..

[78] Singh B., Singh V., Krishnan A., Koshy K., Martinez J.A., Cheng C., Almquist C., Zochodne D.W. (2014). Regeneration of diabetic axons is enhanced by selective knockdown of the pten gene. Brain.

[79] Kaur N., Wohlhueter A.L., Halvorsen S.W. (2002). Activation and inactivation of signal transducers and activators of transcription by ciliary neurotrophic factor in neuroblastoma cells. Cell. Signal..

[80] Smith P.D., Sun F., Park K.K., Cai B., Wang C., Kuwako K., Martinez-Carrasco I., Connolly L., He Z. (2009). Socs3 deletion promotes optic nerve regeneration in vivo. Neuron.

[81] Sun F., Park K.K., Belin S., Wang D., Lu T., Chen G., Zhang K., Yeung C., Feng G., Yankner B.A. (2011). Sustained axon regeneration induced by co-deletion of pten and socs3. Nature.

[82] Belin S., Nawabi H., Wang C., Tang S., Latremoliere A., Warren P., Schorle H., Uncu C., Woolf C.J., He Z. (2015). Injury-induced decline of intrinsic regenerative ability revealed by quantitative proteomics. Neuron.

[83] Marrero M.B., Banes-Berceli A.K., Stern D.M., Eaton D.C. (2006). Role of the jak/stat signaling pathway in diabetic nephropathy. Am. J. Physiol. Renal. Physiol..

[84] Dudley A.C., Thomas D., Best J., Jenkins A. (2005). A vegf/jak2/stat5 axis may partially mediate endothelial cell tolerance to hypoxia. Biochem. J..

[85] Liu Y., Xiao J., Zhao Y., Zhao C., Yang Q., Du X., Wang X. (2020). Microrna-216a protects against human retinal microvascular endothelial cell injury in diabetic retinopathy by suppressing the nos2/jak/stat axis. Exp. Mol. Pathol..

[86] Galvao J., Iwao K., Apara A., Wang Y., Ashouri M., Shah T.N., Blackmore M., Kunzevitzky N.J., Moore D.L., Goldberg J.L. (2018). The kruppel-like factor gene target dusp14 regulates axon growth and regeneration. Investig. Ophthalmol. Vis. Sci..

[87] Lu Y., Brommer B., Tian X., Krishnan A., Meer M., Wang C., Vera D.L., Zeng Q., Yu D., Bonkowski M.S. (2020). Reprogramming to recover youthful epigenetic information and restore vision. Nature.

[88] Au N.P.B., Chand R., Kumar G., Asthana P., Tam W.Y., Tang K.M., Ko C.C., Ma C.H.E. (2022). A small molecule m1 promotes optic nerve regeneration to restore target-specific neural activity and visual function. Proc. Natl. Acad. Sci. USA.

[89] Li Y., Andereggen L., Yuki K., Omura K., Yin Y., Gilbert H.Y., Erdogan B., Asdourian M.S., Shrock C., de Lima S. (2017). Mobile zinc increases rapidly in the retina after optic nerve injury and regulates ganglion cell survival and optic nerve regeneration. Proc. Natl. Acad. Sci. USA.

[90] Au N.P.B., Kumar G., Asthana P., Gao F., Kawaguchi R., Chang R.C.C., So K.F., Hu Y., Geschwind D.H., Coppola G. (2022). Clinically relevant small-molecule promotes nerve repair and visual function recovery. NPJ Regen Med.

[91] Fischer A.J., Bongini R. (2010). Turning muller glia into neural progenitors in the retina. Mol. Neurobiol..

[92] Fausett B.V., Goldman D. (2006). A role for alpha1 tubulin-expressing muller glia in regeneration of the injured zebrafish retina. J. Neurosci..

[93] Goldman D. (2014). Muller glial cell reprogramming and retina regeneration. Nat. Rev. Neurosci..

[94] Hoang T., Wang J., Boyd P., Wang F., Santiago C., Jiang L., Yoo S., Lahne M., Todd L.J., Jia M. (2020). Gene regulatory networks controlling vertebrate retinal regeneration. Science.

[95] Todd L., Hooper M.J., Haugan A.K., Finkbeiner C., Jorstad N., Radulovich N., Wong C.K., Donaldson P.C., Jenkins W., Chen Q. (2021). Efficient stimulation of retinal regeneration from muller glia in adult mice using combinations of proneural bhlh transcription factors. Cell Rep..

[96] Jorstad N.L., Wilken M.S., Grimes W.N., Wohl S.G., VandenBosch L.S., Yoshimatsu T., Wong R.O., Rieke F., Reh T.A. (2017). Stimulation of functional neuronal regeneration from muller glia in adult mice. Nature.

[97] Ueki Y., Wilken M.S., Cox K.E., Chipman L., Jorstad N., Sternhagen K., Simic M., Ullom K., Nakafuku M., Reh T.A. (2015). Transgenic expression of the proneural transcription factor ascl1 in muller glia stimulates retinal regeneration in young mice. Proc. Natl. Acad. Sci. USA.

[98] Xiao D., Jin K., Qiu S., Lei Q., Huang W., Chen H., Su J., Xu Q., Xu Z., Gou B. (2021). In vivo regeneration of ganglion cells for vision restoration in mammalian retinas. Front. Cell Dev. Biol..

[99] Coughlin B.A., Feenstra D.J., Mohr S. (2017). Muller cells and diabetic retinopathy. Vis. Res..

[100] Tran N.M., Shekhar K., Whitney I.E., Jacobi A., Benhar I., Hong G., Yan W., Adiconis X., Arnold M.E., Lee J.M. (2019). Single-cell profiles of retinal ganglion cells differing in resilience to injury reveal neuroprotective genes. Neuron.

[101] Rheaume B.A., Jereen A., Bolisetty M., Sajid M.S., Yang Y., Renna K., Sun L., Robson P., Trakhtenberg E.F. (2018). Single cell transcriptome profiling of retinal ganglion cells identifies cellular subtypes. Nat. Commun..

[102] Sanes J.R., Masland R.H. (2015). The types of retinal ganglion cells: Current status and implications for neuronal classification. Annu. Rev. Neurosci..

[103] Kerrigan-Baumrind L.A., Quigley H.A., Pease M.E., Kerrigan D.F., Mitchell R.S. (2000). Number of ganglion cells in glaucoma eyes compared with threshold visual field tests in the same persons. Investig. Ophthalmol. Vis. Sci..

[104] Robinson G.A., Madison R.D. (2004). Axotomized mouse retinal ganglion cells containing melanopsin show enhanced survival, but not enhanced axon regrowth into a peripheral nerve graft. Vis. Res..

[105] Li L., Fang F., Feng X., Zhuang P., Huang H., Liu P., Liu L., Xu A.Z., Qi L.S., Cong L. (2022). Single-cell transcriptome analysis of regenerating rgcs reveals potent glaucoma neural repair genes. Neuron.

[106] Tapia M.L., Nascimento-Dos-Santos G., Park K.K. (2022). Subtype-specific survival and regeneration of retinal ganglion cells in response to injury. Front. Cell Dev. Biol..

[107] Li S., Yang C., Zhang L., Gao X., Wang X., Liu W., Wang Y., Jiang S., Wong Y.H., Zhang Y. (2016). Promoting axon regeneration in the adult cns by modulation of the melanopsin/gpcr signaling. Proc. Natl. Acad. Sci. USA.

[108] Bray E.R., Yungher B.J., Levay K., Ribeiro M., Dvoryanchikov G., Ayupe A.C., Thakor K., Marks V., Randolph M., Danzi M.C. (2019). Thrombospondin-1 mediates axon regeneration in retinal ganglion cells. Neuron.

[109] Fernandez D.C., Sande P.H., de Zavalia N., Belforte N., Dorfman D., Casiraghi L.P., Golombek D., Rosenstein R.E. (2013). Effect of experimental diabetic retinopathy on the non-image-forming visual system. Chronobiol. Int..

[110] Kumar S., Zhuo L. (2011). Quantitative analysis of pupillary light reflex by real-time autofluorescent imaging in a diabetic mouse model. Exp. Eye Res..

[111] Ba-Ali S., Brondsted A.E., Andersen H.U., Jennum P., Lund-Andersen H. (2020). Pupillary light responses in type 1 and type 2 diabetics with and without retinopathy. Acta Ophthalmol..

[112] Obara E.A., Hannibal J., Heegaard S., Fahrenkrug J. (2017). Loss of melanopsin-expressing retinal ganglion cells in patients with diabetic retinopathy. Investig. Ophthalmol. Vis. Sci..

[113] Feigl B., Zele A.J., Fader S.M., Howes A.N., Hughes C.E., Jones K.A., Jones R. (2012). The post-illumination pupil response of melanopsin-expressing intrinsically photosensitive retinal ganglion cells in diabetes. Acta Ophthalmol..

[114] Wang S., Gottlieb J.L., Sorenson C.M., Sheibani N. (2009). Modulation of thrombospondin 1 and pigment epithelium-derived factor levels in vitreous fluid of patients with diabetes. Arch Ophthalmol..

[115] Jacobi A., Tran N.M., Yan W., Benhar I., Tian F., Schaffer R., He Z., Sanes J.R. (2022). Overlapping transcriptional programs promote survival and axonal regeneration of injured retinal ganglion cells. Neuron.

[116] Kowluru R.A., Mishra M. (2017). Regulation of matrix metalloproteinase in the pathogenesis of diabetic retinopathy. Prog. Mol. Biol. Transl. Sci..

[117] Lobanovskaya N., Jurgenson M., Aonurm-Helm A., Zharkovsky A. (2018). Alterations in the polysialylated neural cell adhesion molecule and retinal ganglion cell density in mice with diabetic retinopathy. Int. J. Ophthalmol..

[118] Hu Z., Mao X., Chen M., Wu X., Zhu T., Liu Y., Zhang Z., Fan W., Xie P., Yuan S. (2022). Single-cell transcriptomics reveals novel role of microglia in fibrovascular membrane of proliferative diabetic retinopathy. Diabetes.

[119] Sun L., Wang R., Hu G., Liu H., Lv K., Duan Y., Shen N., Wu J., Hu J., Liu Y. (2021). Single cell rna sequencing (scrna-seq) deciphering pathological alterations in streptozotocin-induced diabetic retinas. Exp. Eye Res..

[120] Niu T., Fang J., Shi X., Zhao M., Xing X., Wang Y., Zhu S., Liu K. (2021). Pathogenesis study based on high-throughput single-cell sequencing analysis reveals novel transcriptional landscape and heterogeneity of retinal cells in type 2 diabetic mice. Diabetes.

[121] Van Hove I., De Groef L., Boeckx B., Modave E., Hu T.T., Beets K., Etienne I., Van Bergen T., Lambrechts D., Moons L. (2020). Single-cell transcriptome analysis of the akimba mouse retina reveals cell-type-specific insights into the pathobiology of diabetic retinopathy. Diabetologia.

[122] Becker K., Klein H., Simon E., Viollet C., Haslinger C., Leparc G., Schultheis C., Chong V., Kuehn M.H., Fernandez-Albert F. (2021). In-depth transcriptomic analysis of human retina reveals molecular mechanisms underlying diabetic retinopathy. Sci. Rep..

[123] Tian F., Cheng Y., Zhou S., Wang Q., Monavarfeshani A., Gao K., Jiang W., Kawaguchi R., Wang Q., Tang M. (2022). Core transcription programs controlling injury-induced neurodegeneration of retinal ganglion cells. Neuron.

[124] Wang X.W., Li Q., Liu C.M., Hall P.A., Jiang J.J., Katchis C.D., Kang S., Dong B.C., Li S., Zhou F.Q. (2018). Lin28 signaling supports mammalian pns and cns axon regeneration. Cell Rep..

[125] Zhang Y., Williams P.R., Jacobi A., Wang C., Goel A., Hirano A.A., Brecha N.C., Kerschensteiner D., He Z. (2019). Elevating growth factor responsiveness and axon regeneration by modulating presynaptic inputs. Neuron.

[126] Williams P.R., Benowitz L.I., Goldberg J.L., He Z. (2020). Axon regeneration in the mammalian optic nerve. Annu. Rev. Vis. Sci..

[127] Li Y., Struebing F.L., Wang J., King R., Geisert E.E. (2018). Different effect of sox11 in retinal ganglion cells survival and axon regeneration. Front. Genet..

[128] Chang K.C., Bian M., Xia X., Madaan A., Sun C., Wang Q., Li L., Nahmou M., Noro T., Yokota S. (2021). Posttranslational modification of sox11 regulates rgc survival and axon regeneration. eNeuro.

[129] Pernet V., Joly S., Dalkara D., Jordi N., Schwarz O., Christ F., Schaffer D.V., Flannery J.G., Schwab M.E. (2013). Long-distance axonal regeneration induced by cntf gene transfer is impaired by axonal misguidance in the injured adult optic nerve. Neurobiol. Dis..

[130] Luo X., Salgueiro Y., Beckerman S.R., Lemmon V.P., Tsoulfas P., Park K.K. (2013). Three-dimensional evaluation of retinal ganglion cell axon regeneration and pathfinding in whole mouse tissue after injury. Exp. Neurol..

[131] Conceicao R., Evans R.S., Pearson C.S., Hanzi B., Osborne A., Deshpande S.S., Martin K.R., Barber A.C. (2019). Expression of developmentally important axon guidance cues in the adult optic chiasm. Investig. Ophthalmol. Vis. Sci..

[132] Thanos S., Puttmann S., Naskar R., Rose K., Langkamp-Flock M., Paulus W. (2004). Potential role of pax-2 in retinal axon navigation through the chick optic nerve stalk and optic chiasm. J. Neurobiol..

[133] Dominguez-Romero M.E., Slater P.G. (2021). Unraveling axon guidance during axotomy and regeneration. Int. J. Mol. Sci..

[134] Cerani A., Tetreault N., Menard C., Lapalme E., Patel C., Sitaras N., Beaudoin F., Leboeuf D., De Guire V., Binet F. (2013). Neuron-derived semaphorin 3a is an early inducer of vascular permeability in diabetic retinopathy via neuropilin-1. Cell Metab..

[135] Ojima T., Takagi H., Suzuma K., Oh H., Suzuma I., Ohashi H., Watanabe D., Suganami E., Murakami T., Kurimoto M. (2006). Ephrina1 inhibits vascular endothelial growth factor-induced intracellular signaling and suppresses retinal neovascularization and blood-retinal barrier breakdown. Am. J. Pathol..

[136] Dakouane-Giudicelli M., Alfaidy N., de Mazancourt P. (2014). Netrins and their roles in placental angiogenesis. Biomed Res. Int..

[137] Yu Y., Zou J., Han Y., Quyang L., He H., Hu P., Shao Y., Tu P. (2015). Effects of intravitreal injection of netrin-1 in retinal neovascularization of streptozotocin-induced diabetic rats. Drug Des. Devel. Ther..

[138] Cao B., Meng X., Fu Y., Liu P., Lun Y., Wang Y. (2017). Neuron-derived netrin-1 and netrin-4 proteins are additional effective targets in diabetic retinopathy beyond vegf. Int. J. Clin. Exp. Pathol..

[139] Lazzara F., Amato R., Platania C.B.M., Conti F., Chou T.H., Porciatti V., Drago F., Bucolo C. (2021). 1alpha,25-dihydroxyvitamin d(3) protects retinal ganglion cells in glaucomatous mice. J. Neuroinflam..

[140] Yasuda H., Terada M., Maeda K., Kogawa S., Sanada M., Haneda M., Kashiwagi A., Kikkawa R. (2003). Diabetic neuropathy and nerve regeneration. Prog. Neurobiol..

[141] Zhang X., Tenerelli K., Wu S., Xia X., Yokota S., Sun C., Galvao J., Venugopalan P., Li C., Madaan A. (2020). Cell transplantation of retinal ganglion cells derived from hescs. Restor. Neurol. Neurosci..

[142] Rabesandratana O., Chaffiol A., Mialot A., Slembrouck-Brec A., Joffrois C., Nanteau C., Rodrigues A., Gagliardi G., Reichman S., Sahel J.A. (2020). Generation of a transplantable population of human ipsc-derived retinal ganglion cells. Front. Cell Dev. Biol..

[143] Venugopalan P., Wang Y., Nguyen T., Huang A., Muller K.J., Goldberg J.L. (2016). Transplanted neurons integrate into adult retinas and respond to light. Nat. Commun..

[144] Luo Z., Chang K.C., Wu S., Sun C., Xia X., Nahmou M., Bian M., Wen R.R., Zhu Y., Shah S. (2022). Directly induced human retinal ganglion cells mimic fetal rgcs and are neuroprotective after transplantation in vivo. Stem Cell Rep..

[145] Wu S., Chang K.C., Nahmou M., Goldberg J.L. (2018). Induced pluripotent stem cells promote retinal ganglion cell survival after transplant. Investig. Ophthalmol. Vis. Sci..

